# How Do Critical Thinking Ability and Critical Thinking Disposition Relate to the Mental Health of University Students?

**DOI:** 10.3389/fpsyg.2021.704229

**Published:** 2021-08-19

**Authors:** Zhiyuan Liu, Shuangshuang Li, Shouwei Shang, Xuezhu Ren

**Affiliations:** School of Education, Huazhong University of Science and Technology, Wuhan, China

**Keywords:** mental health, critical thinking ability, critical thinking disposition, impulsivity, depression

## Abstract

Theories of psychotherapy suggest that human mental problems associate with deficiencies in critical thinking. However, it currently remains unclear whether both critical thinking skill and critical thinking disposition relate to individual differences in mental health. This study explored whether and how the critical thinking ability and critical thinking disposition of university students associate with individual differences in mental health in considering impulsivity that has been revealed to be closely related to both critical thinking and mental health. Regression and structural equation modeling analyses based on a Chinese university student sample (*N* = 314, 198 females, *M* age = 18.65) revealed that critical thinking skill and disposition explained a unique variance of mental health after controlling for impulsivity. Furthermore, the relationship between critical thinking and mental health was mediated by motor impulsivity (acting on the spur of the moment) and non-planning impulsivity (making decisions without careful forethought). These findings provide a preliminary account of how human critical thinking associate with mental health. Practically, developing mental health promotion programs for university students is suggested to pay special attention to cultivating their critical thinking dispositions and enhancing their control over impulsive behavior.

## Introduction

Although there is no consistent definition of critical thinking (CT), it is usually described as “purposeful, self-regulatory judgment that results in interpretation, analysis, evaluation, and inference, as well as explanations of the evidential, conceptual, methodological, criteriological, or contextual considerations that judgment is based upon” (Facione, 1990, p. 2). This suggests that CT is a combination of skills and dispositions. The skill aspect mainly refers to higher-order cognitive skills such as inference, analysis, and evaluation, while the disposition aspect represents one's consistent motivation and willingness to use CT skills (Dwyer, 2017). An increasing number of studies have indicated that CT plays crucial roles in the activities of university students such as their academic performance (e.g., Ghanizadeh, 2017; Ren et al., 2020), professional work (e.g., Barry et al., 2020), and even the ability to cope with life events (e.g., Butler et al., 2017). An area that has received less attention is how critical thinking relates to impulsivity and mental health. This study aimed to clarify the relationship between CT (which included both CT skill and CT disposition), impulsivity, and mental health among university students.

### Relationship Between Critical Thinking and Mental Health

Associating critical thinking with mental health is not without reason, since theories of psychotherapy have long stressed a linkage between mental problems and dysfunctional thinking (Gilbert, 2003; Gambrill, 2005; Cuijpers, 2019). Proponents of cognitive behavioral therapy suggest that the interpretation by people of a situation affects their emotional, behavioral, and physiological reactions. Those with mental problems are inclined to bias or heuristic thinking and are more likely to misinterpret neutral or even positive situations (Hollon and Beck, 2013). Therefore, a main goal of cognitive behavioral therapy is to overcome biased thinking and change maladaptive beliefs *via* cognitive modification skills such as objective understanding of one's cognitive distortions, analyzing evidence for and against one's automatic thinking, or testing the effect of an alternative way of thinking. Achieving these therapeutic goals requires the involvement of critical thinking, such as the willingness and ability to critically analyze one's thoughts and evaluate evidence and arguments independently of one's prior beliefs. In addition to theoretical underpinnings, characteristics of university students also suggest a relationship between CT and mental health. University students are a risky population in terms of mental health. They face many normative transitions (e.g., social and romantic relationships, important exams, financial pressures), which are stressful (Duffy et al., 2019). In particular, the risk increases when students experience academic failure (Lee et al., 2008; Mamun et al., 2021). Hong et al. (2010) found that the stress in Chinese college students was primarily related to academic, personal, and negative life events. However, university students are also a population with many resources to work on. Critical thinking can be considered one of the important resources that students are able to use (Stupple et al., 2017). Both CT skills and CT disposition are valuable qualities for college students to possess (Facione, 1990). There is evidence showing that students with a higher level of CT are more successful in terms of academic performance (Ghanizadeh, 2017; Ren et al., 2020), and that they are better at coping with stressful events (Butler et al., 2017). This suggests that that students with higher CT are less likely to suffer from mental problems.

Empirical research has reported an association between CT and mental health among college students (Suliman and Halabi, 2007; Kargar et al., 2013; Yoshinori and Marcus, 2013; Chen and Hwang, 2020; Ugwuozor et al., 2021). Most of these studies focused on the relationship between CT disposition and mental health. For example, Suliman and Halabi (2007) reported that the CT disposition of nursing students was positively correlated with their self-esteem, but was negatively correlated with their state anxiety. There is also a research study demonstrating that CT disposition influenced the intensity of worry in college students either by increasing their responsibility to continue thinking or by enhancing the detached awareness of negative thoughts (Yoshinori and Marcus, 2013). Regarding the relationship between CT ability and mental health, although there has been no direct evidence, there were educational programs examining the effect of teaching CT skills on the mental health of adolescents (Kargar et al., 2013). The results showed that teaching CT skills decreased somatic symptoms, anxiety, depression, and insomnia in adolescents. Another recent CT skill intervention also found a significant reduction in mental stress among university students, suggesting an association between CT skills and mental health (Ugwuozor et al., 2021).

The above research provides preliminary evidence in favor of the relationship between CT and mental health, in line with theories of CT and psychotherapy. However, previous studies have focused solely on the disposition aspect of CT, and its link with mental health. The ability aspect of CT has been largely overlooked in examining its relationship with mental health. Moreover, although the link between CT and mental health has been reported, it remains unknown how CT (including skill and disposition) is associated with mental health.

### Impulsivity as a Potential Mediator Between Critical Thinking and Mental Health

One important factor suggested by previous research in accounting for the relationship between CT and mental health is impulsivity. Impulsivity is recognized as a pattern of action without regard to consequences. Patton et al. (1995) proposed that impulsivity is a multi-faceted construct that consists of three behavioral factors, namely, non-planning impulsiveness, referring to making a decision without careful forethought; motor impulsiveness, referring to acting on the spur of the moment; and attentional impulsiveness, referring to one's inability to focus on the task at hand. Impulsivity is prominent in clinical problems associated with psychiatric disorders (Fortgang et al., 2016). A number of mental problems are associated with increased impulsivity that is likely to aggravate clinical illnesses (Leclair et al., 2020). Moreover, a lack of CT is correlated with poor impulse control (Franco et al., 2017). Applications of CT may reduce impulsive behaviors caused by heuristic and biased thinking when one makes a decision (West et al., 2008). For example, Gregory (1991) suggested that CT skills enhance the ability of children to anticipate the health or safety consequences of a decision. Given this, those with high levels of CT are expected to take a rigorous attitude about the consequences of actions and are less likely to engage in impulsive behaviors, which may place them at a low risk of suffering mental problems. To the knowledge of the authors, no study has empirically tested whether impulsivity accounts for the relationship between CT and mental health.

This study examined whether CT skill and disposition are related to the mental health of university students; and if yes, how the relationship works. First, we examined the simultaneous effects of CT ability and CT disposition on mental health. Second, we further tested whether impulsivity mediated the effects of CT on mental health. To achieve the goals, we collected data on CT ability, CT disposition, mental health, and impulsivity from a sample of university students. The results are expected to shed light on the mechanism of the association between CT and mental health.

## Method

### Participants and Procedure

A total of 314 university students (116 men) with an average age of 18.65 years (*SD* = 0.67) participated in this study. They were recruited by advertisements from a local university in central China and majoring in statistics and mathematical finance. The study protocol was approved by the Human Subjects Review Committee of the Huazhong University of Science and Technology. Each participant signed a written informed consent describing the study purpose, procedure, and right of free. All the measures were administered in a computer room. The participants were tested in groups of 20–30 by two research assistants. The researchers and research assistants had no formal connections with the participants. The testing included two sections with an interval of 10 min, so that the participants had an opportunity to take a break. In the first section, the participants completed the syllogistic reasoning problems with belief bias (SRPBB), the Chinese version of the California Critical Thinking Skills Test (CCSTS-CV), and the Chinese Critical Thinking Disposition Inventory (CCTDI), respectively. In the second session, they completed the Barrett Impulsivity Scale (BIS-11), Depression Anxiety Stress Scale-21 (DASS-21), and University Personality Inventory (UPI) in the given order.

### Measures

#### Measures of Critical Thinking Ability

The Chinese version of the California Critical Thinking Skills Test was employed to measure CT skills (Lin, 2018). The CCTST is currently the most cited tool for measuring CT skills and includes analysis, assessment, deduction, inductive reasoning, and inference reasoning. The Chinese version included 34 multiple choice items. The dependent variable was the number of correctly answered items. The internal consistency (Cronbach's α) of the CCTST is 0.56 (Jacobs, 1995). The test–retest reliability of CCTST-CV is 0.63 (*p* < 0.01) (Luo and Yang, 2002), and correlations between scores of the subscales and the total score are larger than 0.5 (Lin, 2018), supporting the construct validity of the scale. In this study among the university students, the internal consistency (Cronbach's α) of the CCTST-CV was 0.5.

The second critical thinking test employed in this study was adapted from the belief bias paradigm (Li et al., 2021). This task paradigm measures the ability to evaluate evidence and arguments independently of one's prior beliefs (West et al., 2008), which is a strongly emphasized skill in CT literature. The current test included 20 syllogistic reasoning problems in which the logical conclusion was inconsistent with one's prior knowledge (e.g., “Premise 1: All fruits are sweet. Premise 2: Bananas are not sweet. Conclusion: Bananas are not fruits.” valid conclusion). In addition, four non-conflict items were included as the neutral condition in order to avoid a habitual response from the participants. They were instructed to suppose that all the premises are true and to decide whether the conclusion logically follows from the given premises. The measure showed good internal consistency (Cronbach's α = 0.83) in a Chinese sample (Li et al., 2021). In this study, the internal consistency (Cronbach's α) of the SRPBB was 0.94.

#### Measures of Critical Thinking Disposition

The Chinese Critical Thinking Disposition Inventory was employed to measure CT disposition (Peng et al., 2004). This scale has been developed in line with the conceptual framework of the California critical thinking disposition inventory. We measured five CT dispositions: truth-seeking (one's objectivity with findings even if this requires changing one's preconceived opinions, e.g., a person inclined toward being truth-seeking might disagree with “I believe what I want to believe.”), inquisitiveness (one's intellectual curiosity. e.g., “No matter what the topic, I am eager to know more about it”), analyticity (the tendency to use reasoning and evidence to solve problems, e.g., “It bothers me when people rely on weak arguments to defend good ideas”), systematically (the disposition of being organized and orderly in inquiry, e.g., “I always focus on the question before I attempt to answer it”), and CT self-confidence (the trust one places in one's own reasoning processes, e.g., “I appreciate my ability to think precisely”). Each disposition aspect contained 10 items, which the participants rated on a 6-point Likert-type scale. This measure has shown high internal consistency (overall Cronbach's α = 0.9) (Peng et al., 2004). In this study, the CCTDI scale was assessed at Cronbach's α = 0.89, indicating good reliability.

#### Measure of Impulsivity

The well-known Barrett Impulsivity Scale (Patton et al., 1995) was employed to assess three facets of impulsivity: non-planning impulsivity (e.g., “I plan tasks carefully”); motor impulsivity (e.g., “I act on the spur of the moment”); attentional impulsivity (e.g., “I concentrate easily”). The scale includes 30 statements, and each statement is rated on a 5-point scale. The subscales of non-planning impulsivity and attentional impulsivity were reversely scored. The BIS-11 has good internal consistency (Cronbach's α = 0.81, Velotti et al., 2016). This study showed that the Cronbach's α of the BIS-11 was 0.83.

#### Measures of Mental Health

The Depression Anxiety Stress Scale-21 was used to assess mental health problems such as depression (e.g., “I feel that life is meaningless”), anxiety (e.g., “I find myself getting agitated”), and stress (e.g., “I find it difficult to relax”). Each dimension included seven items, which the participants were asked to rate on a 4-point scale. The Chinese version of the DASS-21 has displayed a satisfactory factor structure and internal consistency (Cronbach's α = 0.92, Wang et al., 2016). In this study, the internal consistency (Cronbach's α) of the DASS-21 was 0.94.

The University Personality Inventory that has been commonly used to screen for mental problems of college students (Yoshida et al., 1998) was also used for measuring mental health. The 56 symptom-items assessed whether an individual has experienced the described symptom during the past year (e.g., “a lack of interest in anything”). The UPI showed good internal consistency (Cronbach's α = 0.92) in a Chinese sample (Zhang et al., 2015). This study showed that the Cronbach's α of the UPI was 0.85.

### Statistical Analyses

We first performed analyses to detect outliers. Any observation exceeding three standard deviations from the means was replaced with a value that was three standard deviations. This procedure affected no more than 5‰ of observations. Hierarchical regression analysis was conducted to determine the extent to which facets of critical thinking were related to mental health. In addition, structural equation modeling with Amos 22.0 was performed to assess the latent relationship between CT, impulsivity, and mental health.

## Results

### Descriptive Statistics and Bivariate Correlations

Table 1 presents descriptive statistics and bivariate correlations of all the variables. CT disposition such as truth-seeking, systematicity, self-confidence, and inquisitiveness was significantly correlated with DASS-21 and UPI, but neither CCTST-CV nor SRPBB was related to DASS-21 and UPI. Subscales of BIS-11 were positively correlated with DASS-21 and UPI, but were negatively associated with CT dispositions.

**Table 1:** Descriptive results and correlations between all measured variables (*N* = 314).

**Measure**	**M**	**SD**	**Max**	**Min**	**1**	**2**	**3**	**4**	**5**	**6**	**7**	**8**	**9**	**10**	**11**
**1. CCTST-CV**	20.13	3.30	28.00	8.00	−										
**2. SRPBB**	11.70	6.44	20.00	0	0.33^**^	−									
**CCTDI**															
3. Truth seeking	41.08	5.26	58.00	19.00	0.07	0.11	−								
4. Analyticity	43.58	5.04	56.00	24.00	0.10	0.13^*^	0.21^**^	−							
5.Systematically	40.71	5.82	59.00	20.00	0.05	0.08	0.50^**^	0.56^**^	−						
6. Self-confidence	39.86	6.38	59.00	26.00	0.13^*^	0.16^**^	0.19^**^	0.64^**^	0.58^**^	−					
7. Inquisitiveness	46.35	5.81	60.00	33.00	0.08	0.04	0.18^**^	0.63^**^	0.58^**^	0.64^**^	−				
**BIS-11**															
8. Attentional	23.97	4.57	37.00	10.00	−0.19^**^	−028^**^	−023^**^	−036^**^	−040^**^	−043^**^	−035^**^	−			
9. Non-planning	24.59	4.87	39.00	10.00	−004	−006	−022^**^	−034^**^	−045^**^	−037^**^	−035^**^	0.67^**^	−		
10. Motor	24.13	4.80	43.00	12.00	−010	−016^**^	−026^**^	−025^**^	−032^**^	−023^**^	−020^**^	0.33^**^	0.25^**^	−	
**11. DASS-21**	16.13	10.10	58.00	0	−009	−002	−018^**^	−011	−020^**^	−015^**^	−012^*^	0.14^*^	0.21^**^	0.35^**^	−
**12. UPI**	9.13	6.75	34.00	0	−002	−008	−030^**^	−013^*^	−034^**^	−014^*^	−014^*^	0.12^*^	0.19^**^	0.21^**^	0.34^**^

* *p < 0.05*;

** *p < 0.01*.

### Regression Analyses

Hierarchical regression analyses were conducted to examine the effects of CT skill and disposition on mental health. Before conducting the analyses, scores in DASS-21 and UPI were reversed so that high scores reflected high levels of mental health. Table 2 presents the results of hierarchical regression. In model 1, the sum of the Z-score of DASS-21 and UPI served as the dependent variable. Scores in the CT ability tests and scores in the five dimensions of CCTDI served as predictors. CT skill and disposition explained 13% of the variance in mental health. CT skills did not significantly predict mental health. Two dimensions of dispositions (truth seeking and systematicity) exerted significantly positive effects on mental health. Model 2 examined whether CT predicted mental health after controlling for impulsivity. The model containing only impulsivity scores (see model-2 step 1 in Table 2) explained 15% of the variance in mental health. Non-planning impulsivity and motor impulsivity showed significantly negative effects on mental health. The CT variables on the second step explained a significantly unique variance (6%) of CT (see model-2 step 2). This suggests that CT skill and disposition together explained the unique variance in mental health after controlling for impulsivity.^1^

**Table 2:** Hierarchical regression models predicting mental health from critical thinking skills, critical thinking dispositions, and impulsivity (*N* = 314).

**Predictor**	**Model-1β**	**Model-2**	
		**Step 1β**	**Step 2β**
**Critical thinking ability**			
1. CCTST-CV	0.02		0.02
2. SRPBB	0.02		0.02
**Critical thinking dispositons**			
1. Truth seeking	0.17^**^		0.14^*^
2. Analyticity	−005		−008
3. Systematically	0.26^**^		0.18^*^
4. Self-confidence	0.02		0.02
5. Inquisitiveness	0.00		0.00
**Impulsivity**			
1. Attentional impulsivity		0.11	0.14
2. Non-planning impulsivity		−024^**^	−018^*^
3. Motor impulsivity		−032^**^	−026^**^
	*R*^2^ = 0.13	*R*^2^ = 0.15	Δ*R* = 0.06
	*F* = 6.72^**^	*F* = 18.59^**^	*F* = 7.96^**^

*CCTST-CV, The Chinese version of the California Critical Thinking Skills Test; SRPBB, Syllogistic Reasoning Problems with Belief Bias*.

* *p < 0.05*.

** *p < 0*.

Structural equation modeling was performed to examine whether impulsivity mediated the relationship between CT disposition (CT ability was not included since it did not significantly predict mental health) and mental health. Since the regression results showed that only motor impulsivity and non-planning impulsivity significantly predicted mental health, we examined two mediation models with either motor impulsivity or non-planning impulsivity as the hypothesized mediator. The item scores in the motor impulsivity subscale were randomly divided into two indicators of motor impulsivity, as were the scores in the non-planning subscale. Scores of DASS-21 and UPI served as indicators of mental health and dimensions of CCTDI as indicators of CT disposition. In addition, a bootstrapping procedure with 5,000 resamples was established to test for direct and indirect effects. Amos 22.0 was used for the above analyses.

**Figure 1:**
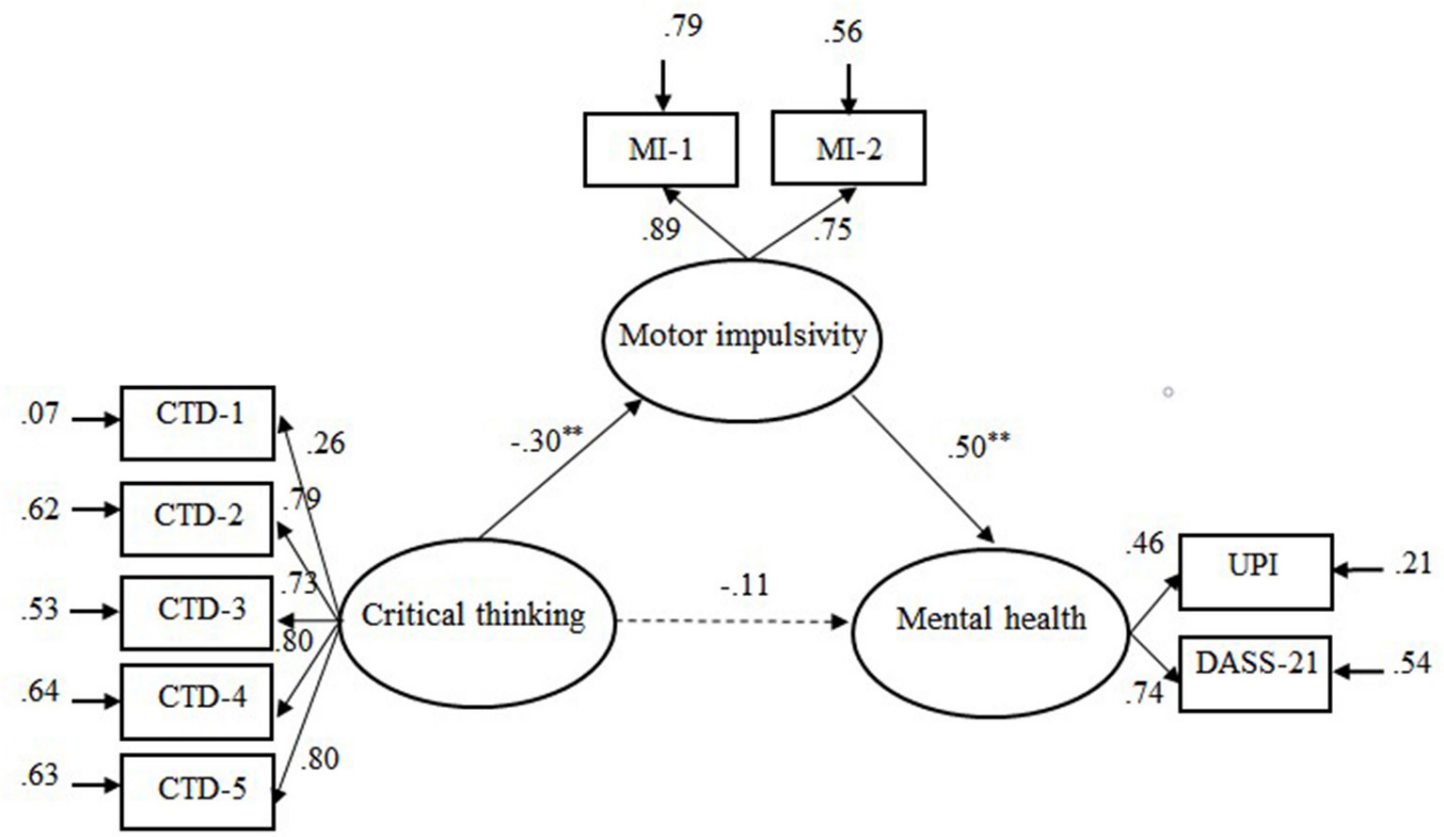
Illustration of the mediation model: Motor impulsivity as mediator variable between critical thinking dispositions and mental health. CTD-l = Truth seeking; CTD-2 = Analyticity; CTD-3 = Systematically; CTD-4 = Self-confidence; CTD-5 = Inquisitiveness. MI-I and MI-2 were sub-scores of motor impulsivity. Solid line represents significant links and dotted line non-significant links. **p < 0.01.

The mediation model that included motor impulsivity (see Figure 1) showed an acceptable fit, $\chi_{\left(23\right)}^{2}$ = 64.71, RMSEA = 0.076, CFI = 0.96, GFI = 0.96, NNFI = 0.93, SRMR = 0.073. Mediation analyses indicated that the 95% boot confidence intervals of the indirect effect and the direct effect were (0.07, 0.26) and (−0.08, 0.32), respectively. As Hayes (2009) indicates, an effect is significant if zero is not between the lower and upper bounds in the 95% confidence interval. Accordingly, the indirect effect between CT disposition and mental health was significant, while the direct effect was not significant. Thus, motor impulsivity completely mediated the relationship between CT disposition and mental health.

**Figure 2:**
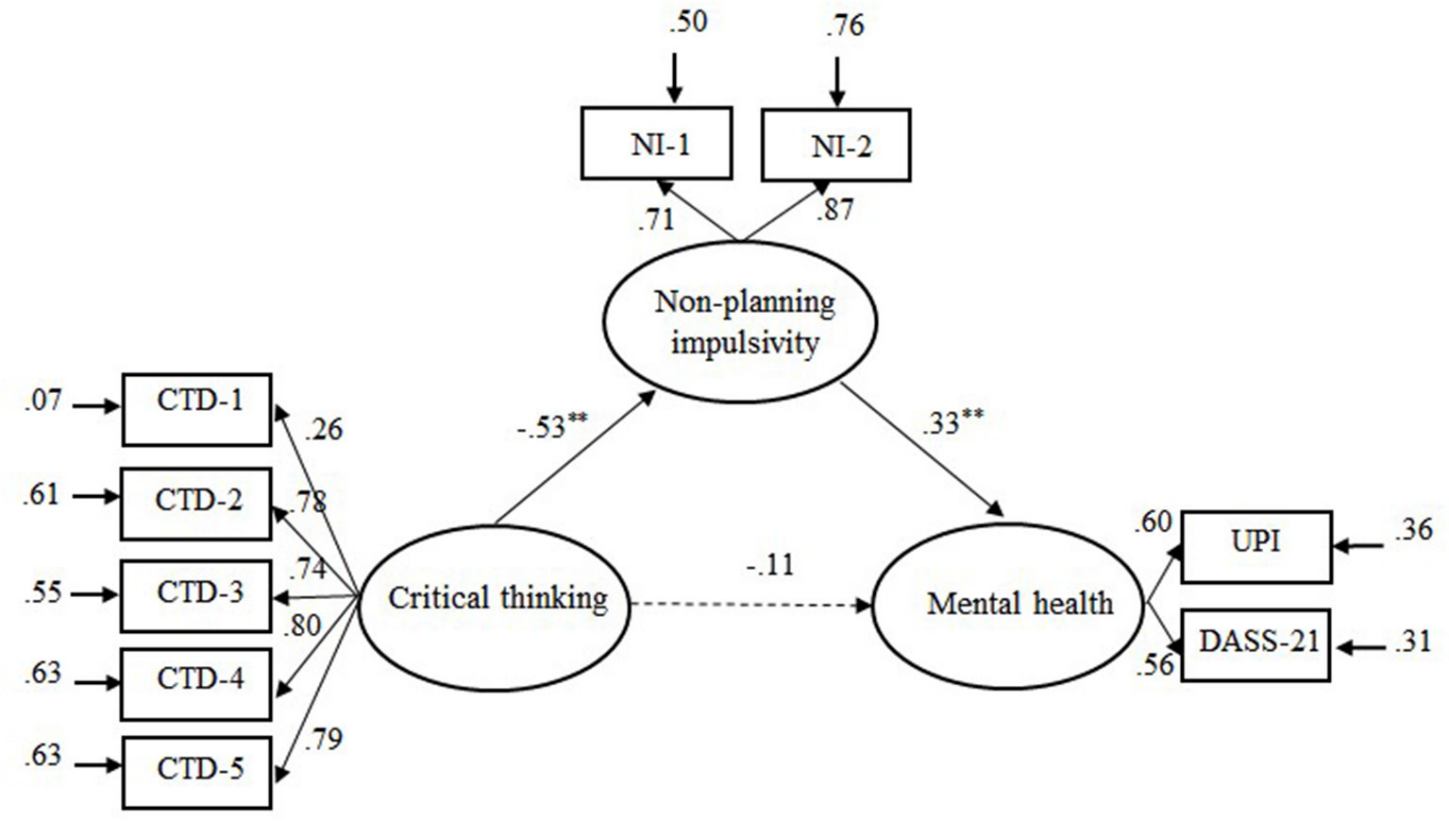
Illustration of the mediation model: Non-planning impulsivity asmediator variable between critical thinking dispositions and mental health. CTD-l = Truth seeking; CTD-2 = Analyticity; CTD-3 = Systematically; CTD-4 = Self-confidence; CTD-5 = Inquisitiveness. NI-I and NI-2 were sub-scores of Non-planning impulsivity. Solid line represents significant links and dotted line non-significant links. **p < 0.01.

The mediation model, which included non-planning impulsivity (see Figure 2), also showed an acceptable fit to the data, $\chi_{\left(23\right)}^{2}$ = 52.75, RMSEA = 0.064, CFI = 0.97, GFI = 0.97, NNFI = 0.95, SRMR = 0.06. The 95% boot confidence intervals of the indirect effect and the direct effect were (0.05, 0.33) and (−0.04, 0.38), respectively, indicating that non-planning impulsivity completely mediated the relationship between CT disposition and mental health.

## Discussion

This study examined how critical thinking skill and disposition are related to mental health. Theories of psychotherapy suggest that human mental problems are in part due to a lack of CT. However, empirical evidence for the hypothesized relationship between CT and mental health is relatively scarce. This study explored whether and how CT ability and disposition are associated with mental health. The results, based on a university student sample, indicated that CT skill and disposition explained a unique variance in mental health. Furthermore, the effect of CT disposition on mental health was mediated by motor impulsivity and non-planning impulsivity. The finding that CT exerted a significant effect on mental health was in accordance with previous studies reporting negative correlations between CT disposition and mental disorders such as anxiety (Suliman and Halabi, 2007). One reason lies in the assumption that CT disposition is usually referred to as personality traits or habits of mind that are a remarkable predictor of mental health (e.g., Benzi et al., 2019). This study further found that of the five CT dispositions, only truth-seeking and systematicity were associated with individual differences in mental health. This was not surprising, since the truth-seeking items mainly assess one's inclination to crave for the best knowledge in a given context and to reflect more about additional facts, reasons, or opinions, even if this requires changing one's mind about certain issues. The systematicity items target one's disposition to approach problems in an orderly and focused way. Individuals with high levels of truth-seeking and systematicity are more likely to adopt a comprehensive, reflective, and controlled way of thinking, which is what cognitive therapy aims to achieve by shifting from an automatic mode of processing to a more reflective and controlled mode.

Another important finding was that motor impulsivity and non-planning impulsivity mediated the effect of CT disposition on mental health. The reason may be that people lacking CT have less willingness to enter into a systematically analyzing process or deliberative decision-making process, resulting in more frequently rash behaviors or unplanned actions without regard for consequences (Billieux et al., 2010; Franco et al., 2017). Such responses can potentially have tangible negative consequences (e.g., conflict, aggression, addiction) that may lead to social maladjustment that is regarded as a symptom of mental illness. On the contrary, critical thinkers have a sense of deliberativeness and consider alternate consequences before acting, and this thinking-before-acting mode would logically lead to a decrease in impulsivity, which then decreases the likelihood of problematic behaviors and negative moods.

It should be noted that although the raw correlation between attentional impulsivity and mental health was significant, regression analyses with the three dimensions of impulsivity as predictors showed that attentional impulsivity no longer exerted a significant effect on mental effect after controlling for the other impulsivity dimensions. The insignificance of this effect suggests that the significant raw correlation between attentional impulsivity and mental health was due to the variance it shared with the other impulsivity dimensions (especially with the non-planning dimension, which showed a moderately high correlation with attentional impulsivity, *r* = 0.67).

Some limitations of this study need to be mentioned. First, the sample involved in this study is considered as a limited sample pool, since all the participants are university students enrolled in statistics and mathematical finance, limiting the generalization of the findings. Future studies are recommended to recruit a more representative sample of university students. A study on generalization to a clinical sample is also recommended. Second, as this study was cross-sectional in nature, caution must be taken in interpreting the findings as causal. Further studies using longitudinal, controlled designs are needed to assess the effectiveness of CT intervention on mental health.

In spite of the limitations mentioned above, the findings of this study have some implications for research and practice intervention. The result that CT contributed to individual differences in mental health provides empirical support for the theory of cognitive behavioral therapy, which focuses on changing irrational thoughts. The mediating role of impulsivity between CT and mental health gives a preliminary account of the mechanism of how CT is associated with mental health. Practically, although there is evidence that CT disposition of students improves because of teaching or training interventions (e.g., Profetto-Mcgrath, 2005; Sanja and Krstivoje, 2015; Chan, 2019), the results showing that two CT disposition dimensions, namely, truth-seeking and systematicity, are related to mental health further suggest that special attention should be paid to cultivating these specific CT dispositions so as to enhance the control of students over impulsive behaviors in their mental health promotions.

## Conclusions

This study revealed that two CT dispositions, truth-seeking and systematicity, were associated with individual differences in mental health. Furthermore, the relationship between critical thinking and mental health was mediated by motor impulsivity and non-planning impulsivity. These findings provide a preliminary account of how human critical thinking is associated with mental health. Practically, developing mental health promotion programs for university students is suggested to pay special attention to cultivating their critical thinking dispositions (especially truth-seeking and systematicity) and enhancing the control of individuals over impulsive behaviors.

## Data Availability Statement

The raw data supporting the conclusions of this article will be made available by the authors, without undue reservation.

## Ethics Statement

The studies involving human participants were reviewed and approved by HUST Critical Thinking Research Center (Grant No. 2018CT012). The patients/participants provided their written informed consent to participate in this study.

## Author Contributions

XR designed the study and revised the manuscript. ZL collected data and wrote the manuscript. SL assisted in analyzing the data. SS assisted in re-drafting and editing the manuscript. All the authors contributed to the article and approved the submitted version.

## Conflict of Interest

The authors declare that the research was conducted in the absence of any commercial or financial relationships that could be construed as a potential conflict of interest.

## Publisher's Note

All claims expressed in this article are solely those of the authors and do not necessarily represent those of their affiliated organizations, or those of the publisher, the editors and the reviewers. Any product that may be evaluated in this article, or claim that may be made by its manufacturer, is not guaranteed or endorsed by the publisher.

## Footnotes

^1^We re-analyzed the data by controlling for age and gender of the participants in the regression analyses. The results were virtually the same as those reported in the study.
